# The Role of Preoperative Abdominal Ultrasound in the Preparation of Patients Undergoing Primary Metabolic and Bariatric Surgery: A Machine Learning Algorithm on 4418 Patients’ Records

**DOI:** 10.1007/s11695-024-07433-9

**Published:** 2024-08-08

**Authors:** Mohamed Hany, Mohamed El Shafei, Mohamed Ibrahim, Ann Samy Shafiq Agayby, Anwar Ashraf Abouelnasr, Moustafa R. Aboelsoud, Ehab Elmongui, Bart Torensma

**Affiliations:** 1 Department of Surgery, Medical Research Institute, Alexandria University, 165 Horreya Avenue, Hadara, 21561 Alexandria Egypt; 2 Madina Women’s Hospital, Alexandria, Egypt; 3 Department of Radiology, Faculty of Medicine, Alexandria University, Alexandria, Egypt; 4 Biomedical Informatics and Medical Statistics, Medical Research Institute, Alexandria University, Alexandria, Egypt; 5 Leiden University Medical Center (LUMC), Leiden, The Netherlands

**Keywords:** Training accuracy, Testing accuracy, Precision, Area under the curve, Ultrasound, Metabolic bariatric surgery, Preoperative evaluation, Magnetic resonance imaging, Computed tomography

## Abstract

**Background:**

The utility of preoperative abdominal ultrasonography (US) in evaluating patients with obesity before metabolic bariatric surgery (MBS) remains ambiguously defined.

**Method:**

Retrospective analysis whereby patients were classified into four groups based on ultrasound results. Group 1 had normal findings. Group 2 had non-significant findings that did not affect the planned procedure. Group 3 required additional or follow-up surgeries without changing the surgical plan. Group 4, impacting the procedure, needed further investigations and was subdivided into 4A, delaying surgery for more assessments, and 4B, altering or canceling the procedure due to critical findings. Machine learning techniques were utilized to identify variables.

**Results:**

Four thousand four hundred eighteen patients’ records were analyzed. Group 1 was 45.7%. Group 2, 35.7%; Group 3, 17.0%; Group 4, 1.5%, Group 4A, 0.8%; and Group 4B, 0.7%, where surgeries were either canceled (0.3%) or postponed (0.4%). The hyperparameter tuning process identified a Decision Tree classifier with a maximum tree depth of 7 as the most effective model. The model demonstrated high effectiveness in identifying patients who would benefit from preoperative ultrasound before MBS, with training and testing accuracies of 0.983 and 0.985. It also showed high precision (0.954), recall (0.962), F1 score (0.958), and an AUC of 0.976.

**Conclusion:**

Our study found that preoperative ultrasound demonstrated clinical utility for a subset of patients undergoing metabolic bariatric surgery. Specifically, 15.9% of the cohort benefited from the identification of chronic calculous cholecystitis, leading to concomitant cholecystectomy. Additionally, surgery was postponed in 1.4% of the cases due to other findings. While these findings indicate a potential benefit in certain cases, further research, including a cost–benefit analysis, is necessary to fully evaluate routine preoperative ultrasound’s overall utility and economic impact in this patient population.

**Graphical Abstract:**

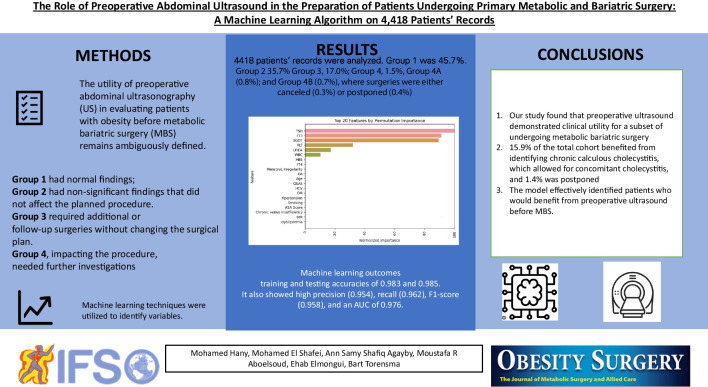

## Introduction

Severe obesity is associated with various medical complications, including a range of gastrointestinal disorders, liver and gallbladder diseases, and other abdominal abnormalities. The utility of preoperative abdominal ultrasonography (US) in evaluating patients with obesity before metabolic bariatric surgery (MBS) remains ambiguously defined.

Some surgeons deem it essential for detecting intra-abdominal anomalies or organ enlargement that could influence outcomes during or post-surgery. Conversely, other specialists consider it unnecessary, arguing that it is time-consuming, not cost-effective, and seldom alters the surgical approach. Furthermore, the effectiveness of US in patients with obesity may be limited due to excessive soft tissue [1–3].

While certain guidelines for MBS recommend abdominal US solely for symptomatic individuals or those with abnormal laboratory findings, others advocate for its routine application in all preoperative assessments [4, 5]. The principal aim of employing the US in patients with obesity is to detect gallbladder and biliary tract issues, given the risk of de novo post-MBS for gallstone disease was 20.7% [6]. Moreover, the rapid weight loss following MBS increases the likelihood of gallstone formation, a concern particularly pertinent in patients undergoing Roux-en-Y gastric bypass (RYGB), where the procedure restricts endoscopic access to the biliary tract in cases of choledocholithiasis [7].

The literature reveals that the value of routine preoperative US screening has been examined, categorizing patients to determine whether to proceed, modify, postpone, or cancel the scheduled surgery. Recommendations for non-routine ultrasound examinations before MBS surgery have also been documented. Nonetheless, these studies fail to disclose the proportion of patients who benefit from such screenings or whether these individuals require concurrent surgical interventions or additional radiological evaluations to aid decision-making processes [1–3, 8, 9].

In our practice, we routinely performed abdominal US on all patients undergoing MBS. Based on these findings, we may consider additional diagnostic methods such as computed tomography (CT) scans, magnetic resonance imaging (MRI), or radiologically guided biopsies to clarify diagnoses further when US results indicate the need for more detailed characterization.

The objective of this study was to conduct a retrospective analysis of patients who underwent primary MBS. We aimed to assess the outcomes of routine preoperative US and explore the role of alternative radiological techniques in cases necessitating further diagnostic evaluation. Moreover, we analyzed how these findings influenced whether to proceed with the surgery as planned, implement modifications, postpone, or cancel the procedure.

Additionally, machine learning techniques were utilized to identify variables with significant predictive capabilities, aiding in developing a clinical prediction model that effectively identifies patients likely to benefit from a preoperative US examination.

## Methods

This retrospective cohort study analyzed medical records of patients who visited the radiology department of the Medical Research Institute, Alexandria University Hospitals, and Madina Women’s Hospital, Alexandria, Egypt, between March 2016 and January 2022 for preoperative evaluation of patients undergoing MBS. The study was approved by the appropriate ethics committee and performed in accordance with the ethical standards of the 1964 Declaration of Helsinki. All patients provided informed consent for the data to be published for research.

### Study Objectives

#### Primary Objective

Patients were categorized into four groups based on ultrasound results. When multiple abnormalities were detected, only the most severe abnormality that had the most significant influence on the procedure and the patient was considered for counting and data analysis.

- ***Group 1*** consisted of patients with normal ultrasound results.
- ***Group 2*** consisted of patients with non-significant findings that did not impact the planned procedure.
- ***Group 3*** consisted of patients with findings that did not affect the surgical plan but required concomitant surgery and/or postoperative follow-up.
- ***Group 4*** consisted of patients with significant findings that directly affected the procedure or required further radiological, laboratory, or endoscopic investigations. Group 4 was then divided into two subgroups:
  - ***Group 4A*** included patients with findings that did not impact the surgical plan but delayed the surgery until other radiological investigations were completed.
  - ***Group 4B*** included patients whose findings directly affected the surgical plan, resulting in either postponement for assessment by another specialty or cancellation of the procedure.

#### Secondary Objective

The study employed machine learning techniques to identify the variables with the highest predictive capabilities. For the construction of a clinical prediction model.

#### Inclusion Criteria

All patients undergoing primary MBS received a preoperative fasting abdominal ultrasound, pelvic, and laboratory testing. Additionally, a multi-disciplinary team (MDT) consisting of a surgeon, internist, dietician, and psychiatrist assessed every patient. However, routine preoperative upper gastrointestinal endoscopy was not performed for all patients during that inclusion time.

#### Exclusion Criteria

Patients undergoing revisional surgery, patients under 18 years of age, and those with incomplete medical records.

### Data Collection

Data collected included age, sex, preoperative body mass index (BMI), laboratory results, sonographic findings, and the subsequent plan.

### Statistical Analysis

Categorical variables were compared using the chi-square test or Fisher’s exact test (for variables with expected frequencies of less than 5 in more than 20% of the contingency tables’ cells). Continuous variables were compared between the four ultrasound outcome groups using analysis of variance (ANOVA); a parametric test was used as the large sample size (4418) supported this, and no severe deviations from normality were observed in Q-Q plots. Additionally, we added a symbol (¥) next to the variables where we used Fisher’s exact test instead of the chi-square test. The significance level was set at 0.05. Statistical analyses were conducted in R (version 4.2.2).

### Prediction Model Development Using Machine Learning

A clinical prediction model was developed using the scikit-learn library (version 1.0.2) in Python. It explored various machine learning algorithms to predict which patients would likely fall into Groups 3 or 4 and for whom ultrasound examination before undergoing bariatric surgery would be beneficial.

The standardized dataset was split into training and test sets in a 70:30 ratio, with 70% of the data allocated to the training set and 30% to the test set. This partitioning enabled the assessment of model performance on unseen data, thus providing insights into the generalization capabilities of the trained models. The classifiers trained included the following algorithms: K-Nearest Neighbors (KNN), Logistic Regression, Support Vector Machine (SVM) with a linear kernel, SVM with a radial basis function (RBF) kernel, Naive Bayes, Decision Tree, Random Forest, Gradient Boosting, and Neural Network. Initially, each classifier was configured with default parameters; then, hyperparameter tuning was conducted through grid search with cross-validation for each classifier.

The grid search process involved evaluating multiple combinations of hyperparameters using a fivefold cross-validation strategy to identify each classifier’s optimal set of hyperparameters. Table 1 shows the hyperparameters included in the grid search process for each algorithm. Following hyperparameter tuning, each classifier was trained on the training dataset using the optimal hyperparameters determined.

**Table 1:** Hyperparameters included in the tuning process with grid search

Classifier	Hyperparameters
KNN	Number of neighbors: 3, 5, and 7
Logistic Regression	C: 0.1, 1, and 10
SVM linear	C: 0.1, 1, and 10
SVM kernalized	C: 0.1, 1, and 10, gamma: 0.1, 1, and 10
Naive Bayes	None
Decision Tree	Maximum tree depth: 3, 5, and 7
Random Forest	Number of trees: 50, 100, and 200
Gradient Boosted	Number of trees: 50, 100, and 201
Neural Network	Number of hidden layers sizes: 10, 50, and 100, activation: relu, tanh

*KNN*, K-Nearest Neighbors; *SVM*, Support Vector Machine

Subsequently, the performance of each classifier was assessed using both the training and test datasets to evaluate their generalization ability. Test accuracy for each classifier was determined as the proportion of correctly classified instances in the test set when using the best hyperparameters. The classifier with the best performance among all algorithms was identified, and the most influential patient characteristics, comorbidities, and lab investigations shaping the predictive capabilities of the best-performing model were analyzed using permutation feature importance methodology. This technique involves permuting the values of each variable and observing the subsequent change in model performance.

With the obtained results, we identified the top 20 variables by sorting the mean importance. Subsequently, we fitted a logistic regression model with Lasso regularization to further refine our feature selection process, aiming to filter the variables with the most significant impact on prediction. Finally, we determined the training and testing accuracy, in addition to the precision, recall, F1 score, and AUC of the logistic regression model using the variables with non-zero coefficients.

## Results

This study analyzed 5720 medical records of patients. After applying the exclusion criteria, 4418 patients’ records were analyzed.

**Fig. 1:**
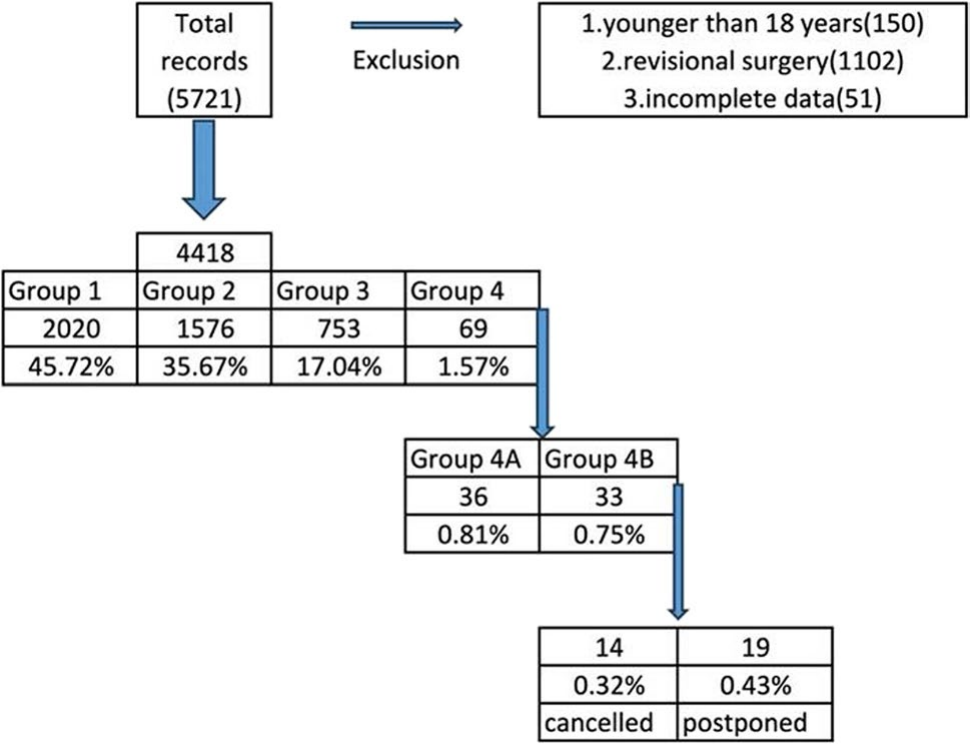
Flowchart of inclusions of patients

Group 1 was the largest at 45.7%. Group 2 comprised 35.7% and had minor, non-impactful findings. Group 3, making up 17.0%, required additional surgery or follow-up without altering the original surgical plan. Group 4, the smallest at 1.5%, included significant findings that affected the procedure; this included Group 4A (0.8%), where additional imaging caused delays, and Group 4B (0.7%), where surgeries were either canceled (0.3%) or postponed (0.4%) (Fig. 1).

### Baseline Characteristics

In total, 70.7% were female, and 29.3% were male. The average age was 43.3 ± 13.8 years. The mean BMI was 48.1 ± 7.5 kg/m^2^.

The most prevalent associated medical problems were osteoarthritis (OA) (11.6%), diabetes mellitus (DM) (9.8%), and dyslipidemia (7.3%). Notably, there were 26 cases of previous hepatitis C virus infection (HCV) (0.6%) (Table 2).

**Table 2:** Patient characteristics, comorbidities, and preoperative laboratory investigations stratified by the results of ultrasound findings

Variable	Total cohort (*N* = 4418)	Group 1 (*n* = 2020)	Group 2 (*n* = 1576)	Group 3 (*n* = 753)	Group 4 (*n* = 69)	*p*
Gender						
Female	3124 (70.7%)	1428 (70.7%)	1161 (73.7%)	476 (63.2%)	59 (85.5%)	< 0.001*
Male	1294 (29.3%)	592 (29.3%)	415 (26.3%)	277 (36.8%)	10 (14.5%)	
Age (years)	43.3 ± 13.8	40.8 ± 13.2	44.6 ± 14.6	47.4 ± 12.8	44.2 ± 11.2	< 0.001*
BMI (kg/m^2^)	48.1 ± 7.5	45.7 ± 6.2	49.3 ± 8.0	52.2 ± 7.7	45.3 ± 5.3	< 0.001*
Smoking	774 (17.5%)	400 (19.8%)	350 (22.2%)	12 (1.6%)	12 (17.4%)	< 0.001*
ASA Score						
1	1547 (35.0%)	707 (35.0%)	552 (35.0%)	264 (35.1%)	24 (34.8%)	
2	1635 (37.0%)	747 (37.0%)	583 (37.0%)	279 (37.1%)	26 (37.7%)	1.000
3	1016 (23.0%)	465 (23.0%)	362 (23.0%)	173 (23.0%)	16 (23.2%)	
4	220 (5.0%)	101 (5.0%)	79 (5.0%)	37 (4.9%)	3 (4.3%)	
Associated medical problems						
Hypertension	112 (2.5%)	15 (0.7%)	56 (3.6%)	31 (4.1%)	10 (14.5%)	< 0.001*
Diabetes	431 (9.8%)	100 (5.0%)	220 (14.0%)	95 (12.6%)	16 (23.2%)	< 0.001*
Dyslipidemia	321 (7.3%)	120 (5.9%)	100 (6.3%)	89 (11.8%)	12 (17.4%)	< 0.001*
Obstructive sleep apnea syndrome¥	33 (0.7%)	10 (0.5%)	15 (1.0%)	3 (0.4%)	5 (7.2%)	< 0.001*
Osteoarthritis	511 (11.6%)	241 (11.9%)	162 (10.3%)	88 (11.7%)	20 (29.0%)	< 0.001*
Menstrual Irregularity¥	72 (1.6%)	55 (2.7%)	12 (0.8%)	3 (0.4%)	2 (2.9%)	< 0.001*
Chronic venous insufficiency¥	83 (1.9%)	60 (3.0%)	15 (1.0%)	5 (0.7%)	3 (4.3%)	< 0.001*
Lab investigations						
HB	13.4 ± 1.2	13.6 ± 1.0	13.5 ± 1.0	12.6 ± 1.6	13.0 ± 0.6	< 0.001*
WBC	8.0 ± 1.5	7.9 ± 1.7	7.9 ± 1.2	8.4 ± 1.2	6.6 ± 2.9	< 0.001*
PLT	276.2 ± 43.6	261.8 ± 30.8	292.0 ± 52.9	286.9 ± 25.7	221.2 ± 81.3	< 0.001*
Urea	28.7 ± 4.9	26.9 ± 4.3	29.3 ± 3.8	32.6 ± 5.6	25.0 ± 6.7	< 0.001*
Creatine	0.8 ± 0.1	0.7 ± 0.1	0.9 ± 0.2	0.7 ± 0.1	0.8 ± 0.1	< 0.001*
FBS	109.5 ± 33.5	121.3 ± 42.3	99.4 ± 11.4	98.2 ± 25.4	114.8 ± 37.6	< 0.001*
HBA1C	5.9 ± 0.4	5.8 ± 0.5	6.0 ± 0.4	5.9 ± 0.1	6.3 ± 0.3	< 0.001*
SGOT	22.1 ± 7.5	17.4 ± 4.8	27.7 ± 7.7	22.8 ± 3.7	21.0 ± 6.5	< 0.001*
SGPT	21.7 ± 6.9	17.5 ± 5.1	25.0 ± 6.3	26.1 ± 5.4	23.7 ± 10.6	< 0.001*
Triglycerides	156.7 ± 52.4	162.4 ± 38.8	141.4 ± 63.1	173.3 ± 49.6	155.8 ± 70.1	< 0.001*
Cholesterol	199.1 ± 38.8	205.8 ± 38.8	185.2 ± 37.5	210.2 ± 29.1	195.5 ± 65.4	< 0.001*
TSH	1.9 ± 0.8	2.0 ± 0.7	2.2 ± 0.9	1.2 ± 0.2	1.4 ± 0.5	< 0.001*
FT3	3.1 ± 0.8	3.4 ± 0.4	2.5 ± 0.8	3.5 ± 1.0	3.3 ± 1.1	< 0.001*
FT4	1.6 ± 1.7	1.1 ± 0.2	2.4 ± 2.6	1.3 ± 0.2	1.4 ± 0.5	< 0.001*
HCV positive	26 (0.6%)	1 (0.0%)	1 (0.1%)	1 (0.1%)	23 (33.3%)	< 0.001*
SHBS positive	1 (0.0%)	1 (0.0%)	0 (0.0%)	0 (0.0%)	0 (0.0%)	1.000

*Statistically significant (*p* < 0.05). ¥ Fisher exact test was used. *BMI*, body mass index; *ASA*, American Society of Anesthesiologists; *HB*, hemoglobin; *WBC*, white blood cell; *PLT*, platelet count; *FBS*, fasting blood sugar; *HBA1C*, hemoglobin A1C; *AST*, aspartate aminotransferase; *ALT*, alanine transaminase; *TSH*, thyroid stimulating hormone; *F3*, free triiodothyronine; *FT4*, free thyroxine; *HCV*, hepatitis C virus; *HBS*, hepatitis B surface antigen; *HCV*, hepatitis C virus

### Ultrasound Examination Findings Stratified by Groups

In Group 2, the most prevalent finding was fatty liver and hepatomegaly, accounting for 87.7% across the group and 31.28% of the total cohort. In Group 3, chronic calculous cholecystitis was the most prevalent condition, representing 93.2% and 15.89% of the total cohort. Finally, in Group 4, the most prevalent conditions were hepatic focal lesions at 20.3% within the group (0.32% from the total cohort), renal lesions at 13.0% (0.20% from the total cohort), and pancreatic lesions at 10.1% (0.16% from the total cohort) (Table 3).

**Table 3:** Ultrasound examination findings stratified by groups

Groups	Findings	*N* = 4418	% from the group (95% CI)	% from the total cohort (95% CI)
Group 1 (*n* = 2020)	No findings	2020	100 (99.78, 100)	45.72 (44.26, 47.19)
Group 2 (*n* = 1576)	Fatty liver and hepatomegaly	1382	87.69 (85.97, 89.22)	31.28 (29.93, 32.66)
	Simple ovarian cyst	52	3.30 (2.52, 4.31)	1.18 (0.90, 1.54)
	Uterine fibroid	27	1.71 (1.17, 2.50)	0.61 (0.42, 0.89)
	Simple renal cysts	26	1.65 (1.12, 2.42)	0.59 (0.40, 0.87)
	Hemangioma single or multiple	16	1.02 (0.62, 1.66)	0.36 (0.22, 0.59)
	Simple liver cysts	16	1.02 (0.62, 1.66)	0.36 (0.22, 0.59)
	Benign prostatic hyperplasia	15	0.95 (0.57, 1.58)	0.34 (0.20, 0.57)
	Focal fatty sparing area	14	0.89 (0.52, 1.51)	0.32 (0.18, 0.54)
	Splenomegaly	9	0.57 (0.29, 1.11)	0.20 (0.10, 0.40)
	Chronic kidney disease	5	0.32 (0.12, 0.77)	0.11 (0.04, 0.28)
	Renal angiomyolipoma < 4 cm	5	0.32 (0.12, 0.77)	0.11 (0.04, 0.28)
	Pelvic congestion and varicosities	4	0.25 (0.08, 0.68)	0.09 (0.03, 0.24)
	Splenosis	3	0.19 (0.04, 0.59)	0.07 (0.01, 0.21)
	Clacified liver granuloma	2	0.13 (0.01, 0.50)	0.05 (0.00, 0.18)
Group 3 (*n* = 753)	Chronic calcular cholecystitis**	702	93.23 (91.18, 94.82)	15.89 (14.84, 17.00)
	Non obstructing urinary tract stones	20	2.66 (1.71, 4.10)	0.45 (0.29, 0.70)
	Paraumbilical hernia ¥	11	1.46 (0.79, 2.64)	0.25 (0.13, 0.45)
	Gall bladder polyps**	7	0.93 (0.42, 1.96)	0.16 (0.07, 0.34)
	Inguinal hernia**	7	0.93 (0.42, 1.96)	0.16 (0.07, 0.34)
	Old splenic infarct ¥	2	0.27 (0.01, 1.04)	0.05 (0.00, 0.18)
	Ovarian dermoid cyst**	2	0.27 (0.01, 1.04)	0.05 (0.00, 0.18)
	Renal angiomyolipoma > 4 cm ¥	2	0.27 (0.01, 1.04)	0.05 (0.00, 0.18)
Group 4 (*n* = 69)*	Hepatic focal lesion	14	20.29 (12.43, 31.41)	0.32 (0.18, 0.54)
	Renal lesion	9	13.04 (6.86, 23.28)	0.20 (0.10, 0.40)
	Pancreatic lesion	7	10.14 (4.79, 19.87)	0.16 (0.07, 0.34)
	Large ovarian cyst	5	7.25 (2.83, 16.34)	0.11 (0.04, 0.28)
	Adrenal lesion	4	5.80 (1.92, 14.52)	0.09 (0.03, 0.24)
	Mesenteric lymph nodes	4	5.80 (1.92, 14.52)	0.09 (0.03, 0.24)
	Abdominal cyst	3	4.35 (1.06, 12.64)	0.07 (0.01, 0.21)
	Splenic lesion	3	4.35 (1.06, 12.64)	0.07 (0.01, 0.21)
	Abdominal wall lesion	2	2.90 (0.26, 10.70)	0.05 (0.00, 0.18)
	Adnexal lesion	2	2.90 (0.26, 10.70)	0.05 (0.00, 0.18)
	Choledochal cyst	2	2.90 (0.26, 10.70)	0.05 (0.00, 0.18)
	Distal CBD stone	2	2.90 (0.26, 10.70)	0.05 (0.00, 0.18)
	Ileitis-appendicitis	2	2.90 (0.26, 10.70)	0.05 (0.00, 0.18)
	Large abdominal lymph node	2	2.90 (0.26, 10.70)	0.05 (0.00, 0.18)
	Ureteric dilatation	2	2.90 (0.26, 10.70)	0.05 (0.00, 0.18)
	Chronic PV thrombosis	1	1.45 (0.00, 8.66)	0.02 (0.00, 0.14)
	Chronic SMV thrombosis	1	1.45 (0.00, 8.66)	0.02 (0.00, 0.14)
	Inguinal lymph node	1	1.45 (0.00, 8.66)	0.02 (0.00, 0.14)
	Large iliac lymph node	1	1.45 (0.00, 8.66)	0.02 (0.00, 0.14)
	Situs ambiguous	1	1.45 (0.00, 8.66)	0.02 (0.00, 0.14)
	Solid adnexal lesion	1	1.45 (0.00, 8.66)	0.02 (0.00, 0.14)

*During the examination for other causes such as pancreatic and renal lesions, two incidental findings were detected. These findings were a gastric gastrointestinal tumor (GIST) and an esophageal leiomyoma, which ultimately resulted in the cancellation of the surgery. **Concomitant surgeries performed. ¥Follow-up. *CBD*, common bile duct stone; *PV*, porta vein; *SMV*, superior mesenteric venous

### Findings that Did Not Directly Influence the Surgical Plan

In Group 4A, findings did not impact the surgical plan but delayed the surgery until other radiological investigations were completed. Pancreatic lesions were further explored by CT and MRI, identifying conditions like ectopic spleen and simple cysts. Abdominal wall lesions were examined via MRI, detecting endometriomas and fibromatosis. Choledochal cysts required magnetic resonance cholangiopancreatography (MRCP), which diagnosed Type IA cysts per Todani’s classification [10]. Hepatic lesions called for triphasic CT and sometimes further tests like ultrasound-guided biopsies, which identified adenomas, hemangiomas, and focal nodular hyperplasia. Renal lesions led to CT scans and biopsies, revealing angiomyolipomas and oncocytomas. Other findings led to extra diagnostics by CT scan, such as adrenal lesions, chronic portal vein thrombosis and superior mesenteric vein (SMV) thrombosis, splenic, adnexal lesions, and mesenteric lymph nodes (Table 4).

**Table 4:** Group 4A’s findings that did not impact the surgical plan but delayed the surgery until other radiological investigations were completed

Lesion	Extra diagnostics testing before surgery decision	Number of diagnostic radiological modalities	Final diagnosis	*n*	% from total 4418 (95% CI)
Pancreatic lesion	CT then MRI of the pancreas	2	Ectopic splenule at tail	1	0.02 (0.00, 0.14)
			Simple cyst	1	0.02 (0.00, 0.14)
Abdominal wall lesion	MRI	1	Endometrioma	1	0.02 (0.00, 0.14)
			Fibromatosis	1	0.02 (0.00, 0.14)
Choledochal cyst	MRCP	1	Type IA cyst(Todani’s classification)	2	0.05 (0.00, 0.18)
Hepatic focal lesion	Triphasic CT	1	Adenoma	2	0.05 (0.00, 0.18)
			Hemangioma	3	0.07 (0.01, 0.21)
			Hyalinized hemangioma	1	0.02 (0.00, 0.14)
			Focal nodular hyperplasia	1	0.02 (0.00, 0.14)
	Triphasic CT followed by US-guided biopsy	2	Atypical focal steatotic nodule	1	0.02 (0.00, 0.14)
Large iliac lymph node	CT followed by US-guided biopsy	2	Granulomatous node	1	0.02 (0.00, 0.14)
Abdominal cyst	CT then US-guided aspiration	2	Mesenteric (Lymphatic cyst)	1	0.02 (0.00, 0.14)
	CT	1	Diaphragmatic mesothelial cyst	1	0.02 (0.00, 0.14)
			Tialgut cyst	1	0.02 (0.00, 0.14)
Renal lesion	CT	1	Angiomyolipoma	1	0.02 (0.00, 0.14)
	CT followed by guided biopsy	2	Oncocytoma	1	0.02 (0.00, 0.14)
Situs ambiguous	CT	1	Polysplenia, right-sided stomach, midline gallbladder	1	0.02 (0.00, 0.14)
Adrenal lesion	CT	1	Adenoma	4	0.09 (0.03, 0.24)
Chronic PV thrombosis	CT	1	Follow-up	1	0.02 (0.00, 0.14)
Chronic SMV thrombosis	CT	1	Follow-up	1	0.02 (0.00, 0.14)
Splenic lesion	CT	1	Hemangioma	2	0.05 (0.00, 0.18)
			Infarct	1	0.02 (0.00, 0.14)
Adnexal lesion	CT	1	pelvic inflammatory disease and multiple ovarian cysts	1	0.02 (0.00, 0.14)
			Calcified large fibroid	1	0.02 (0.00, 0.14)
Mesenteric lymph nodes	CT	1	Mesenteric panniculitis	2	0.05 (0.00, 0.18)
			Diverticulosis	2	0.05 (0.00, 0.18)

*CT*, computed tomography; *MRI*, magnetic resonance imaging; *US*, ultrasound; *MRCP*, magnetic resonance cholangiopancreatography

### Findings that Directly Influenced the Surgical Plan

Group 4B directly affected the surgical plan, resulting in either postponement or cancellation of the procedure.

In total, 19 cases (0.4% total cohort, 57.6% of this group) were postponed, with a range of final diagnoses varied from several cysts, presents of stones, suspected Crohn’s disease, sarcoidosis, or serous cystadenoma.

In 14 cases (0.3% of the total cohort, 42.4% of this group), the MBS procedure was canceled because the range of final diagnoses varied from esophageal leiomyoma to non-Hodgkin lymphoma, several liver metastases, and tumors (ovarian, pancreatic, intraductal papillary-mucinous, and lymphoma) (Table 5).

**Table 5:** Group 4B findings directly affected the surgical plan, resulting in either postponement for assessment by another specialty or cancellation of the procedure

Lesion	Extra diagnostics testing before surgery decision	Number of diagnostic radiological modalities	Final diagnosis	Decision	*n*	% from total 4418 (95% CI)
Renal lesion	CT followed by US-guided aspiration	2	Renal cyst Bosniak III	Postponed	1	0.02 (0.00, 0.14)
	CT	1	Hydronephrosis	Postponed	5	0.11 (0.04, 0.28)
			Kidney free: esophageal leiomyoma	Cancelled*	1	0.02 (0.00, 0.14)
Ureteric dilatation	CT	1	Distal stone	Postponed	2	0.05 (0.00, 0.18)
Ileitis-appendicitis	CT	2	Suspected Crohn’s disease	Postponed	2	0.05 (0.00, 0.18)
Large abdominal lymph node	CT and CT-guided biopsy	2	NHL (retro pancreatic node)	Cancelled	1	0.02 (0.00, 0.14)
			Sarcoidosis	Postponed	1	0.02 (0.00, 0.14)
Inguinal lymph node	CT and CT-guided biopsy	2	NHL	Cancelled	1	0.02 (0.00, 0.14)
Distal CBD stone	MRCP	1	CBD stone (floating)	Postponed	2	0.05 (0.00, 0.18)
Hepatic lesion	CT and percutaneous aspiration	2	Hydatid cyst	Postponed	1	0.02 (0.00, 0.14)
	CT followed by US-guided biopsy	2	Cystic metastasis (ovary)	Cancelled	1	0.02 (0.00, 0.14)
			Metastases (colon, breast)	Cancelled	4	0.09 (0.03, 0.24)
Pancreatic lesion	CT and MRI of the pancreas	2	Intraductal papillary-mucinous tumor (IPMT)	Cancelled	2	0.05 (0.00, 0.18)
	CT and MRI of the pancreas followed by guided biopsy	3	Neuroendocrine tumor (NET)	Cancelled	2	0.05 (0.00, 0.18)
	CT	1	Pancreas free: gastric GIST	Cancelled*	1	0.02 (0.00, 0.14)
Solid adnexal lesion	MRI of the pelvis	1	Ovarian tumor	Cancelled	1	0.02 (0.00, 0.14)
Large ovarian cyst	MRI of the pelvis	1	Hemorrhagic endometriotic cyst	Postponed	2	0.05 (0.00, 0.18)
	MRI then US-guided aspiration	2	Simple cyst	Postponed	2	0.05 (0.00, 0.18)
			Serous cystadenoma	Postponed	1	0.02 (0.00, 0.14)

*During further examination for other causes such as pancreatic and renal lesions, two incidental findings were detected. These findings were a gastric gastrointestinal tumor (GIST) and an esophageal leiomyoma, which ultimately resulted in the cancellation of the surgery. *CT*, computed tomography; *MRI*, magnetic resonance imaging; *US*, ultrasound; *NHL*, non-Hodgkin lymphoma; *IPMT*, intraductal papillary-mucinous tumor; *NET*, neuroendocrine tumor; *CBD*, common bile duct

### Comparison Analysis Between Group 4A and Group 4B

Both groups predominantly consisted of females, with no significant differences in gender distribution (*p* = 0.693). Group 4B patients were older and had higher BMIs than those in Group 4A (*p* < 0.001 for age and BMI). There were no significant differences in smoking prevalence between the groups (*p* = 0.155). A comparison of American Society of Anesthesiologists (ASA) scores indicated a shift towards higher scores in Group 4B, with no patients scoring 1, compared to 66.7% in Group 4A. Group 4B had 48.5% of patients with an ASA score of 3 and 9.1% with a score of 4. Associated medical problems like hypertension, DM, and dyslipidemia showed no significant differences, except for a higher prevalence of obstructive sleep apnea syndrome (OSAS) in Group 4B (*p* = 0.021). Laboratory parameters varied significantly between the groups, although fasting blood sugar (FBS), hemoglobin A1C (HBA1C), cholesterol, and triglyceride levels were similar. Additionally, previous hepatitis C infection prevalence was significantly greater in Group 4A (*p* < 0.001) (Table 6).

**Table 6:** Comparison between Group 4A and Group 4B in terms of patients’ characteristics, comorbidities, and preoperative lab investigations

Characteristics	Group 4A (*n* = 36)	Group 4B (*n* = 33)	*p*
Gender			
Female	30 (83.3%)	29 (87.9%)	0.693
Male	6 (16.7%)	4 (12.1%)	
Age (years)	35.4 ± 7.5	53.8 ± 4.9	< 0.001*
BMI (kg/m^2^)	41.6 ± 2.9	49.5 ± 4.0	< 0.001*
Smoking	9 (25.0%)	3 (9.1%)	0.155
ASA Score			
1	24 (66.7%)	0 (0.0%)	
2	12 (33.3%)	14 (42.4%)	< 0.001*
3	0 (0.0%)	16 (48.5%)	
4	0 (0.0%)	3 (9.1%)	
Associated medical problems			
Hypertension	5 (13.9%)	5 (15.2%)	1.000
DM	9 (25.0%)	7 (21.2%)	0.931
Dyslipidemia	5 (13.9%)	7 (21.2%)	0.629
OSAS	0 (0.0%)	5 (15.2%)	0.021*
OA	11 (30.6%)	9 (27.3%)	0.972
Menstrual irregularity	2 (5.6%)	0 (0.0%)	0.494
Chronic venous insufficiency	1 (2.8%)	2 (6.1%)	0.603
Lab investigations			
HB	13.5 ± 0.4	12.5 ± 0.5	< 0.001*
WBC	5.7 ± 2.7	7.4 ± 2.9	0.013*
PLT	266.3 ± 88.7	172.1 ± 26.1	< 0.001*
Urea	28.6 ± 7.3	21.1 ± 2.5	< 0.001*
Creatine	0.8 ± 0.0	0.8 ± 0.1	0.514
FBS	106.7 ± 27.1	123.7 ± 45.1	0.067
HbA1C	6.3 ± 0.2	6.2 ± 0.4	0.344
SGOT	22.4 ± 5.9	19.4 ± 6.9	0.056
SGPT	28.5 ± 8.6	18.4 ± 10.1	< 0.001*
Triglycerides	161.6 ± 61.7	149.5 ± 78.8	0.482
Cholesterol	186.4 ± 52.5	205.5 ± 76.8	0.238
TSH	1.7 ± 0.5	1.1 ± 0.1	< 0.001*
FT3	4.0 ± 0.4	2.5 ± 1.1	< 0.001*
FT4	1.0 ± 0.2	1.7 ± 0.5	< 0.001*
HCV	23 (63.9%)	0 (0.0%)	< 0.001*
HBS	0 (0.0%)	0 (0.0%)	

*Statistically significant (*p* < 0.05). *BMI*, body mass index; *ASA*, American Society of Anesthesiologists; *HB*, hemoglobin; *WBC*, white blood cell; *PLT*, platelet count; *FBS*, fasting blood sugar; *HBA1C*, hemoglobin A1C; *AST*, aspartate aminotransferase; *ALT*, alanine transaminase; *TSH*, thyroid stimulating hormone; *F3*, free triiodothyronine; *FT4*, free thyroxine; *HCV*, hepatitis C virus; *HBS*, hepatitis B surface antigen

### Concomitant Surgeries

Concomitant surgeries, performed alongside the primary procedure, addressed various conditions after US diagnosis: chronic calculous cholecystitis (*n* = 702, 93.2%), gallbladder polyps (*n* = 7, 0.9%), ovarian dermoid cysts (*n* = 2, 0.3%), and inguinal hernias (*n* = 7, 0.9%), which were found in Group 3.

### Clinical Prediction Model

**Fig. 2:**
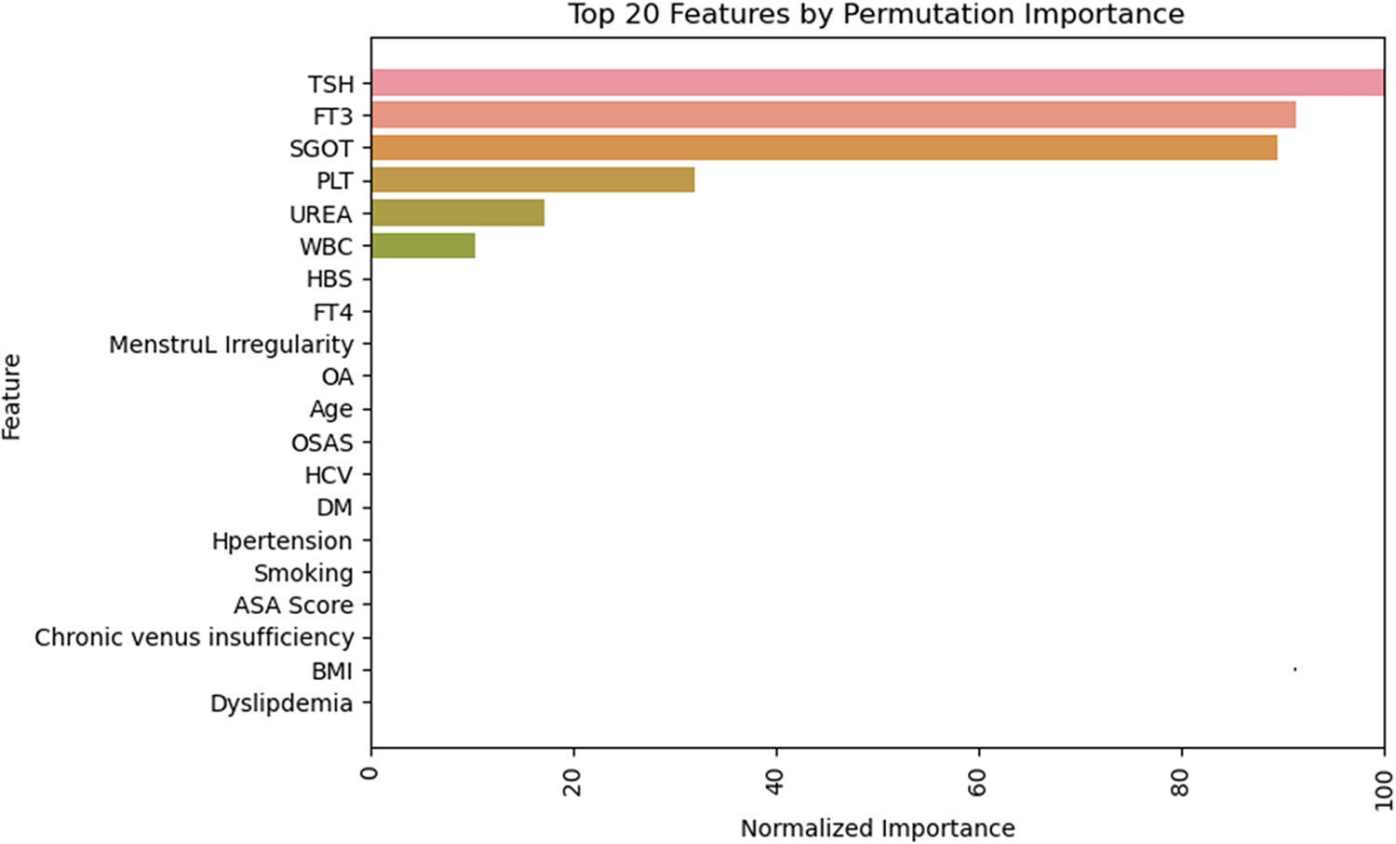
Shaping the predictive capabilities of the best-performing model (Decision Tree classifier). Features importance for the Decision Tree classifier. Permutation variable importance for the most accurate model (Decision Tree) represents the decrease in the model score when each variable is shuffled randomly to break its relationship with the outcome indicating its importance in prediction. Scores were normalized to a maximum of 100 to facilitate comparison

The hyperparameter tuning process identified a Decision Tree classifier with a maximum tree depth of 7 as the most effective model. Figure 2 illustrates the top 20 variables that enhanced this model’s predictive power, determined using the permutation feature importance technique.

Logistic regression with Lasso regularization, utilizing these top variables, identified several with non-zero coefficients—BMI, diabetes, smoking, HCV previous infection, WBC, FT4, platelet count, AST, and TSH—subsequently incorporated into the final logistic regression model. This model achieved training and testing accuracies of 0.983 and 0.985, respectively. The model also showed high precision (0.954), recall (0.962), F1 score (0.958), and an area under the curve (AUC) of 0.976, confirming efficacy in accurately identifying patients in Groups 3 or 4 who would benefit from an ultrasound examination before MBS (Table 7, Fig. 2).

**Table 7:** Results of the grid search process with hyperparameter tuning

Classifier	Best hyperparameters	Training accuracy	Test accuracy
KNN	Number of neighbors: 3	0.99	0.994
Logistic regression	C: 10	0.997	0.997
SVM linear	C: 1	0.998	0.999
SVM kernalized	C: 10, gamma: 0.1	0.989	0.995
Naive Bayes	None	0.825	0.753
Decision Tree	Maximum tree depth: 7	1.000	1.000
Random Forest	Number of trees: 50	1.000	1.000
Gradient Boosted	Number of trees: 51	0.999	1.000
Neural Network	Number of hidden layers sizes: 10, activation: tanh	0.996	0.996

*KNN*, K-Nearest Neighbors; *SVM*, Support Vector Machine

## Discussion

In this retrospective cohort study, 4418 patients’ records were analyzed whereby mandatory ultrasonography (US) examinations were performed in the radiology department before primary metabolic bariatric surgery (MBS). Four groups were defined: Group 1, which consisted of patients with no US findings, and Group 4, which had significant findings that directly affected the procedure or required further radiological, laboratory, or endoscopic investigations.

Additionally, a clinical prediction model was developed by exploring various machine learning algorithms to predict which patients would likely fall into Group 3 (findings that did not affect the surgical plan but required concomitant surgery and/or postoperative follow-up), or Group 4.

To the best of our knowledge, several published studies have examined the role of the US and its value in routine preoperative screening. These studies categorized patients into different groups, with the goal of either maintaining or canceling the scheduled surgery. Additionally, they recommended non-routine ultrasound examinations before surgery. However, none of these studies showed the percentage of patients who benefited from this screening. It remains unclear whether these patients underwent simultaneous surgery or additional radiological examinations to support decision-making [1–3, 8, 9].

### Routine Use of the Ultrasound

The routine use of the US in preoperative evaluations for MBS patients is debated concerning cost-effectiveness and clinical relevance. Some guidelines from decades ago (2008 and 2013) recommend the US only for symptomatic patients, while others endorse its universal use [5, 11]. In the updated guideline for 2019, the US is still advised for symptomatic patients, with assigned grades “weak” and “no conclusive evidence and/or expert opinion” as evidence, whereby no added literature was found in the updated guidelines 2019 [4].

Our study aims to enhance the discussion within the field of MBS by determining which patients with obesity would benefit from preoperative US and identifying those for whom it may not be necessary.

### Radiological Evaluation Before MBS

In our study, no pertinent findings were identified via the US in 45.7% of the cases within our Group A. So, 54.3% of all patients have a relevant finding in the US. In 17.0% of cases, these findings did not alter the surgical plan but necessitated concomitant surgery and/or postoperative monitoring. Nevertheless, only 1.5% of the total cohort was affected where findings directly influenced the surgical procedure or necessitated additional radiological, laboratory, or endoscopic investigations; this proportion is relatively small, raising statistical concerns regarding the adequacy of these figures for calculations such as the number needed to treat and the balance of benefits versus costs and resource use for the hospital.

On the other hand, within this small subgroup—representing 0.3% of the total cohort—procedures were canceled due to the detection of multiple tumors and metastases. Despite the cancellations, early detection still proved beneficial for the patients involved. A study by Lesourd et al. also tested preoperative screening, in this case with CT scans, for diagnosing malignancy in patients undergoing MBS. The results were that the CT scan could not be recommended for cancer screening before MBS, as this study only identified malignancies in 0.6% of cases [6]. Another study by Yu et al. investigated CT and MRI scans before MBS to exclude malignant diseases. However, this approach proved also unsuccessful [12]. Our studies (*n* = 13) have identified results concerning malignancies, similar to those of both studies. It remains uncertain whether failing to detect these conditions would have resulted in greater patient harm.

A recent 2023 study sought to investigate the utility of preoperative CT/MRI in predicting postoperative complications. It examined variables such as the ratios of visceral fat to muscle mass. These metrics provide crucial perioperative information for predicting which patients will likely develop complications after surgery [12]. Other studies found excessive visceral fat and low muscle mass are linked to 30-day complications following abdominal surgery [13, 14]. Additionally, a high visceral adipose tissue-to-skeletal muscle ratio is associated with increased risks of postoperative fistula and bleeding after procedures like gastrectomy or pancreaticoduodenectomy [15]. Radiology assessments could have a role in the prediction. Nevertheless, none has been validated in a sufficient external cohort until now.

### Concomitant Surgery

As discussed, preoperative screening in the US could be an invaluable and indispensable part of the pre-MBS workup, given its relative ease of performance and safety (no radiation, no invasive intervention, no need for sedation/anesthesia). Beyond discovering pathologies that may alter the operative plan, pre-MBS US could address three critical issues: the presence of stones in the biliary tree, the status of the liver, and the existence of abdominal wall hernias. These aspects underscore the comprehensive utility of ultrasound in optimizing surgical planning and patient outcomes.

Our study identified high concomitant surgeries (CS) rates, particularly for chronic calculous cholecystitis (*n* = 702, 93.2%). From this perspective, preoperative ultrasound (US) assessment could inform the surgeon of the necessity for CS, thereby facilitating better preparation for the patient, reducing the costs associated with potential additional operations post-primary MBS, and considering the 20.7% risk of de novo gallstone disease post-MBS as a realistic concern [6]. Moreover, a systematic review of CS associated with MBS indicated that mortality rates were comparable to those without CS. However, the complication rates were slightly higher in the CS group (odds ratio [OR] 1.2, 95% confidence interval [CI] 1.1–1.3) [16]. Therefore, CS are generally safe and enhance surgical preparedness preoperatively, which reopens the debate regarding the mandatory use of US.

### Detection and Grading of MAFLD Using Preoperative Ultrasound

Metabolic-associated fatty liver disease (MAFLD) is highly prevalent among patients with obesity, making its assessment a crucial component of the preoperative evaluation for MBS. In Group 2, the most prevalent finding was fatty liver and hepatomegaly, accounting for 87.7% across the group and 31.28% of the total cohort. The current literature supports the role of ultrasound in detecting MAFLD. Studies highlight the effectiveness of ultrasound in identifying hepatic steatosis and differentiating it from more advanced stages of liver disease. Furthermore, incorporating elastography as a diagnostic tool improves diagnostic accuracy, enabling the detection of significant fibrosis that might impact surgical outcomes and long-term patient health [17, 18].

MBS has been rigorously investigated as a treatment for MAFLD over the past decade, with a substantial body of evidence emerging from systematic reviews (SR) [19–25]. For instance, an SR conducted in 2022 demonstrated that MBS’s effectiveness in reducing the resolution of steatosis was improved in 56% of patients, ballooning degeneration in 49%, inflammation in 45%, and fibrosis in 25% [24]. This reflects the generally favorable outcomes associated with MBS in managing MAFLD. When reviewing the literature, none of the studies mentioned any form of postponing the surgery or the effect on the surgical plan due to the grade of MAFLD. Nevertheless, this makes sense, as the SR by Zhou had exclusion criteria stating, “Exclusion criteria were as follows: (1) patients with cirrhosis or a history of liver transplants.”

A review by Geerts et al. on MBS for non-alcoholic fatty liver disease found and confirmed that weight loss is the cornerstone in the treatment of MAFLD. Still, it is difficult to achieve and maintain long-term target goals with conservative lifestyle changes alone. Patients with obesity and MAFLD fibrosis could benefit from MBS. Evidence shows that MBS is safe, improves steatosis, inflammation, and fibrosis scores, and reduces mortality risk from cardiovascular disease and MAFLD-associated HCC. Other SRs confirm these findings. However, patients with cirrhosis (Grade 4 type) need to be carefully selected by a multi-disciplinary team of specialists to assess the risk and determine the appropriate type of surgery [26].

This highlights the dilemma that most studies do not select patients due to the associated risks, underscoring the need for thorough preoperative assessment in a multi-disciplinary team (MDT) setting to formulate the best surgical plan after US testing.

### Detection of Abdominal Wall Hernias

In our study, the preoperative ultrasound detected hernias in 0.9% of the patients. While not all cases resulted in concomitant surgery, this early detection facilitated individual surgical planning and allowed for timely intervention in select cases. Though a smaller percentage, the detection of hernias underscores the utility of preoperative ultrasound in providing a comprehensive assessment that contributes to the overall surgical strategy. Something that was confirmed by a study by Young et al. that concluded that ultrasound is a valuable diagnostic tool for managing patients with unclear diagnoses of abdominal wall hernias. The findings from ultrasound can significantly influence therapeutic decisions, enabling more efficient and cost-effective treatment by expediting clinical management [27].

### Machine Learning

Nevertheless, it remains challenging to identify which patients benefit from preoperative US. The dilemma often revolves around balancing the costs against the health benefits derived from the understanding that you can diagnose and treat patients. In our study, we also explored the development of machine learning (ML) models. This advancement could provide significant support in the following steps, potentially enhancing our ability to make informed decisions and improving patient outcomes through more precise and predictive analytics.

Our ML model has identified several key predictive variables: BMI, diabetes status, smoking habits, HCV previous infection, WBC count, FT4 levels, platelet count, AST activity, and TSH levels. Correlations based on data that are “invisible to the naked eye,” whereby such insights could advance our understanding by identifying underlying variables that are predictors in workup processes, guiding when the US would be a logical next step in specific clinical presentations. However, external validation of these results is necessary to determine if these characteristics are consistent across other studies.

ML offers significant benefits in clinical research, particularly in enhancing diagnostic accuracy and personalizing treatment approaches. By leveraging large datasets, ML can uncover complex patterns and relationships that may not be apparent through traditional statistical methods, thus offering more profound insights into disease mechanisms and patient responses.

In MBS, ML primarily predicts postoperative outcomes such as weight loss and complications. A systematic review by Bektaş et al. highlighted that these algorithms could predict postoperative complications and weight loss with as high as 98% accuracy [28]. However, the study primarily concentrated on postoperative metrics rather than preoperative diagnostics, and the lack of external validation remains a significant constraint, limiting the broader applicability of these findings. Two reviews on artificial intelligence (AI) and ML highlighted that the heterogeneity of current studies demonstrates the need for meticulous validation, strict reporting systems, and reliable benchmarking to ensure the clinical validity of future research. These models have shown remarkable results, aiding physicians in the decision-making process, thus improving the quality of care and contributing to precision medicine. However, several legal and ethical hurdles must be addressed before these methods can be routinely used in clinical practice [29, 30]. So, ML could revolutionize predictive healthcare by enabling more precise and earlier interventions, ultimately reducing costs and improving outcomes. Further integration of ML with real-time data from electronic health records could lead to dynamic models that adjust to new information, improving their predictive accuracy over time. This ongoing evolution will likely foster more collaborative research and potentially lead to preventive medicine and patient management breakthroughs.

Protecting patient data privacy is essential when using machine learning (ML) in healthcare. Key measures include data anonymization, secure data storage and transfer, and compliance with regulations like GDPR and HIPAA. Data should be anonymized to remove personally identifiable information (PII), encryption methods should safeguard data throughout its lifecycle, and legal frameworks must guide data usage and consent. ML algorithms can perpetuate biases present in training data, leading to unfair outcomes. Key considerations include ensuring diverse and inclusive training data, enhancing transparency through explainable AI (XAI), and regularly auditing and updating models to maintain accuracy and relevance.

ML in clinical settings should align with medical ethics, focusing on beneficence, non-maleficence, justice, and autonomy. Models should benefit patients, minimize harm, ensure equitable access, and respect patient autonomy through informed consent and transparency.

Addressing these ethical considerations ensures that ML applications in healthcare are effective, equitable, and trustworthy, aligning with the principles of ethical medical practice.

### Potential Confounding Factors and How They Might Have Influenced the Study Outcomes

Our study focuses on individuals with obesity, but several factors could still introduce confounding effects. Religion and cultural practices affect our cohort, where alcohol consumption is absent due to religious beliefs. This contrasts with other groups where alcohol impacts liver health and surgical outcomes. While age was accounted for, ethnicity remains critical. Our model tested on a specific ethnic group needs validation across diverse populations.

Associated medical conditions like diabetes, hypertension, and cardiovascular diseases can influence risk profiles and outcomes, introducing confounders. Our study’s retrospective nature may have missed some relevant information. Other potential confounders include variations in laboratory parameters, such as microRNAs affecting gene expression and recovery, and operator variability among ultrasound technicians and radiologists. Differences in surgical techniques and surgeons’ experience can also impact outcomes, confounding the association between preoperative ultrasound findings and surgical success.

### Limitations

The study has several limitations. First, the findings may be influenced by operator dependency, as it was conducted across two university hospitals, where operator experience and technique variations could affect outcomes. Second, the maturity of the machine learning algorithms could also be a limitation, as initial applications of these technologies may not fully capture complex clinical nuances, which could impact the accuracy and reliability of predictions. Third, due to the study’s retrospective nature, correcting all potential confounding factors across the patient population was impossible, which might have influenced the results. This highlights the need for further research to address the impacts of operator dependency, the evolution of machine learning techniques in medical settings, and the control of confounding variables in retrospective analyses.

Ultrasound imaging is highly operator-dependent, with the technician’s skill and experience influencing image quality and accuracy. Variability in training and interpretation among technicians and radiologists can lead to inconsistencies in detecting and classifying conditions. Less experienced operators may overlook or misinterpret findings, while more experienced operators provide more accurate assessments, introducing bias and affecting reliability.

Different ultrasound machines and settings can also produce variations in image quality. Equipment calibration, transducer types, and imaging protocols impact results, leading to inconsistencies when comparing data across different sites or within the same institution over time.

Future studies should standardize ultrasound protocols and provide extensive operator training to mitigate these limitations. Incorporating automated or semi-automated image analysis software could reduce operator dependency and improve consistency and accuracy.

In summary, ultrasound’s operator-dependent nature and variability in imaging techniques are limitations that can affect study outcomes. Acknowledging these factors underscores the need for standardization and training to enhance the reliability of ultrasound assessments.

## Conclusion

Our study found that preoperative ultrasound demonstrated clinical utility for a subset of patients undergoing metabolic bariatric surgery. Specifically, 15.9% of the cohort benefited from the identification of chronic calculous cholecystitis, leading to concomitant cholecystectomy. Additionally, surgery was postponed in 1.4% of the cases due to other findings. While these findings indicate a potential benefit in certain cases, further research, including a cost–benefit analysis, is necessary to fully evaluate routine preoperative ultrasound’s overall utility and economic impact in this patient population. Future research should focus on developing a refined clinical prediction model incorporating key predictive variables from this study to improve its accuracy and clinical utility.

## Data Availability

Data available with the corresponding author.
